# Skeletal muscle metabolic characteristics and fresh meat quality defects associated with wooden breast

**DOI:** 10.3389/fphys.2024.1501362

**Published:** 2024-10-30

**Authors:** Linnea A. Rimmer, Morgan D. Zumbaugh

**Affiliations:** Department of Animal Sciences and Industry, Kansas State University, Manhattan, KS, United States

**Keywords:** wooden breast, myopathy, poultry, skeletal muscle metabolism, fresh meat quality

## Abstract

Wooden breast (WB) is a myopathy that occurs in pectoralis major (PM) muscles, predominately affecting large, fast-growing broilers. Severe myodegeneration, increased hypoxia, reduced blood flow, and increased collagen deposition are hallmark characteristics of WB that culminate in unsatisfactory fresh meat quality attributes, such as poor water-holding capacity, tenderness, and processing characteristics. Therefore, WB meat is often downgraded resulting in economic losses for the United States poultry industry. Although WB has been well characterized, its etiology remains undefined. As the scientific community continues to resolve mechanisms responsible for WB onset, understanding biochemical changes associated with WB may facilitate solutions to negate its poor meat quality attributes. Given changes in metabolism of living muscle can alter biochemical processes during the conversion of muscle to meat, this review aims to summarize and discuss the current knowledge of WB muscle and meat biochemistry. For example, it appears metabolic pathways that support combating stress are upregulated in WB muscle at the expense of glycolytic flux, which presumably contributes to the high ultimate pH of WB meat. Further, perturbed function of WB mitochondria, such as altered calcium handling, impacts aspects of postmortem metabolism and proteolysis. Collectively, metabolic dysfunction of WB muscle alters the biochemical processes that occur during the conversion of muscle to meat, and thus contributes to the poor WB meat quality.

## 1 Introduction

Poultry is the fastest growing sector of the meat industry in the United States (Interagency Agricultural Projections Committee, 2020), largely because of its affordable price and positive nutritional benefits. Rising global demand for high-quality poultry products has driven the U.S. poultry industry to nearly double production from 2001 to 2021 (Miller et al., 2022). To accomplish this feat, the industry has implemented genetic selection and nutritional advancements to achieve superior broiler growth rates (Baker et al., 1999; Kidd et al., 2001; National Chicken Council, 2022). For example, broilers in 1970 were an average of 3.62 lbs at market after approximately 56 days, whereas broilers in 2023 took approximately 47 days to reach an average of 6.54 lbs at market (National Chicken Council, 2022). While the industry improved production efficiency and yields, an unexpected increase in the prevalence of myopathies accompanied these advancements. In fact, fast growth rate is associated with an increase in WB prevalence, and affected broilers can begin presenting symptoms of the pathology as early as 2 weeks of age (Sihvo et al., 2014; Sihvo et al., 2017). Affected filets are characterized as being palpably hard to the touch, pale in color, and having occasional white striping that results in low acceptance by consumers due to poor texture and usability of the meat (Kuttappan et al., 2012; Mudalal et al., 2015; Xing et al., 2020). Although the etiology of WB largely remains undefined, many factors have been identified that contribute to the onset and progression of the myopathy. This review aims to summarize the current knowledge of the underlying muscle physiology and fresh meat quality defects associated with the WB myopathy.

## 2 Skeletal muscle metabolic characteristics associated with WB

**FIGURE 1:**
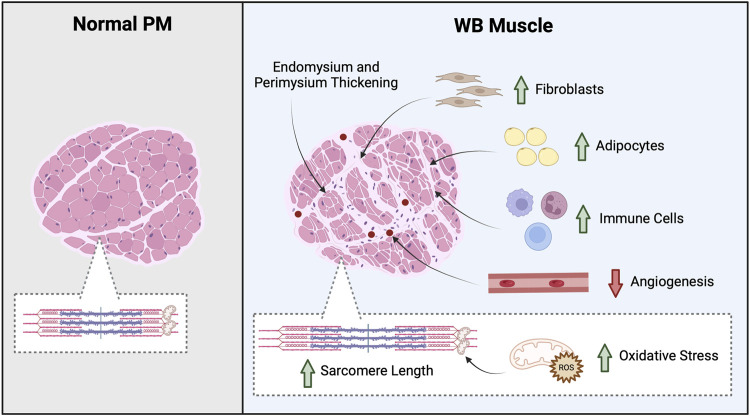
Overview of WB tissue morphology. Greater myodegeneration and an increase in fibroblast, adipocyte, and immune cells suggests WB muscle coordinates a response to repair the damaged tissue; however, non-muscle cells infiltrate the tissue. For example, an increase in macrophages and neutrophils as well as thicker endomysium and perimysium connective tissue layers are found in WB muscle. In addition, WB muscles often exhibit an increase in oxidative stress and decrease angiogenesis. Created in BioRender.

Skeletal muscle is composed of a heterogeneous population of muscle fibers that can be classified by metabolism (oxidative or glycolytic) and contractile speed (slow or fast). While slow fibers (type I) rely heavily on oxidative metabolism, presumably to fuel long or continuous bouts of work, fast fibers (type IIA, IIX, and IIB) vary in their metabolic capability and can range from having a high oxidative capacity to a high glycolytic capacity. Composition of muscle fibers varies within muscles and determines overall muscle phenotype as well as susceptibility to disease (Talbot and Maves, 2016). Pectoralis major muscles of broilers are nearly 100% type IIB fibers, which are characterized as highly glycolytic with a fast contraction speed (Smith and Fletcher, 1988; Roy et al., 2006; Verdiglione and Cassandro, 2013a; Hosotani et al., 2021). Indeed, PM muscles of healthy broilers exhibit low mitochondrial content, high glycolytic capacity, and thus can function under anaerobic conditions (Dransfield and Sosnicki, 1999; Verdiglione and Cassandro, 2013b; Du et al., 2017; Huo et al., 2022). In WB muscle, insufficient angiogenesis during periods of rapid growth results in diminished vascularization and hypoxic conditions (Mutryn et al., 2015; Thanatsang et al., 2020). In fact, an increase in gene expression and protein abundance of hypoxia-inducible factor 1 (HIF-1α; transcription factor that mediates cellular responds to low oxygen levels) as well as an increase in associated proteins involved in the hypoxic response supports the notion of a limited oxygen environment in WB muscle (Greene et al., 2019; Greene et al., 2020). Although highly glycolytic muscle fibers of PM muscles should be able to accommodate a reduced oxygen environment (Minchenko et al., 2002; Kierans and Taylor, 2021), WB muscle exhibits metabolic dysfunction (Abasht et al., 2016; Kuttappan et al., 2017a; Hosotani et al., 2020; Hasegawa et al., 2022; Carvalho et al., 2023; Wang et al., 2023). Indeed, an increase in reactive oxygen species (ROS) and markers of cellular stress are often reported in WB muscle (Figure 1) (Hasegawa et al., 2022; Carvalho et al., 2023; Wang et al., 2023). While WB muscle also upregulates scavenging pathways to combat stress (Hasegawa et al., 2022; Carvalho et al., 2023; Wang et al., 2023), WB muscle is not able to counteract the onset of disease.

### 2.1 Overview of glycolysis and its ancillary pathways

After glucose enters myofibers, hexokinase phosphorylates glucose to generate glucose-6-phoshate (G-6-P), which can be used for energy storage, energy production, supporting anabolic pathways, or as a precursor for metabolic signaling depending on cellular needs. For example, G-6-P is used to synthesize glycogen during times of energy surplus or to fuel glycolysis during times of energy scarcity (Rao et al., 2015; Matarneh et al., 2018b). Alternatively, G-6-P can be shunted into the pentose phosphate pathway (PPP) to generate NADPH for lipid synthesis and scavenging ROS or to produce five-carbon precursors for nucleotide synthesis (Stincone et al., 2015). In addition, G-6-P can be converted to fructose-6-phosphate (F-6-P) and shunted into the hexosamine biosynthetic pathway (HBP) to synthesize UDP-GlcNAc, which is used by the enzyme O-GlcNAc transferase (OGT) to catalyze the O-linked addition of β-N-acetyl glucosamine to target proteins (Hart and Akimoto, 2009). One of the most abundant post-translational modifications, O-GlcNAcylation regulates over 4,000 target proteins in response to cellular nutrient availability (Ma and Hart, 2014). While the HBP only consumes 2%–5% of cellular glucose utilization, O-GlcNAcylation has widespread implications on skeletal muscle metabolism (Marshall et al., 1991; Bond and Hanover, 2015). The multiple fates of glycolytic intermediates contribute to the plastic nature and metabolic flexibility of healthy skeletal muscle.

#### 2.1.1 Glycolytic flux

**FIGURE 2:**
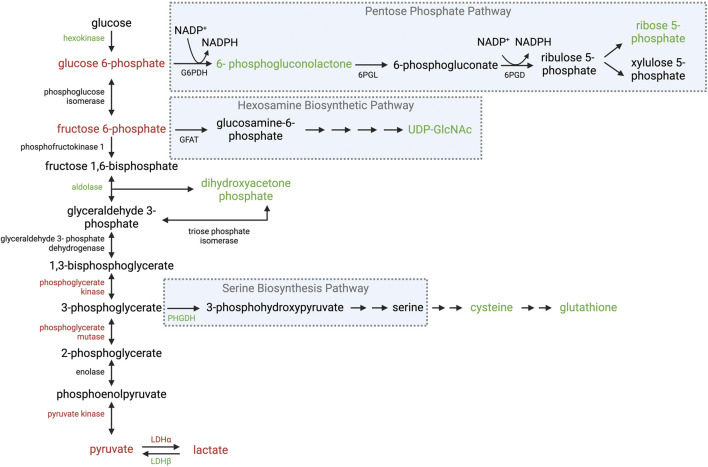
Overview of glycolysis and its ancillary pathways in WB muscle. Schematic of enzymatic and intermediate changes in glycolysis, the pentose phosphate pathway, hexosamine biosynthetic pathway, and serine biosynthesis pathway observed in WB muscle. Red text indicates a decrease in gene expression and/or protein abundance of the enzyme or intermediate. Green text indicates an increase in gene expression and/or protein abundance of the enzyme or intermediate. Black text was not evaluated or no changes were reported. Abbreviations: G6PDH, glucose-6-phosphate dehydrogenase; 6PGL, 6-phosphogluconolactonase; 6PGD, 6-phosphogluconate dehydrogenase; GFAT, glutamine fructose-6-phosphate aminotransferase; PHGDH, phosphoglycerate dehydrogenase; LDHα, lactate dehydrogenase alpha; LDHβ lactate dehydrogenase beta. Created in BioRender.

Given the highly glycolytic nature of broiler PM muscles, it is reasonable to assume WB muscles would adapt to hypoxic conditions associated with the myopathy. However, WB muscles exhibit reduced levels of glycogen, G-6-P, and F-6-P suggesting WB decreases glycolytic flux (Abasht et al., 2016; Baldi et al., 2020). This notion is supported by diminished levels of the glycolytic end products pyruvate and lactate in WB muscle (Abasht et al., 2016). Further, protein abundance of several enzymes in the latter half of glycolysis are also decreased in WB muscle including phosphoglycerate kinase, phosphoglycerate mutase, and pyruvate kinase (Abasht et al., 2016; Soglia et al., 2016; Kuttappan et al., 2017a; Carvalho et al., 2023). The final glycolytic intermediate, pyruvate, can either be converted to lactate through lactate dehydrogenase alpha (LDHα) or enter the tricarboxylic acid (TCA) cycle through pyruvate dehydrogenase (PDH) or pyruvate carboxylase (PC). Glycolytic type IIB fibers predominately divert pyruvate through LDHα to produce lactate, which is then shuttled out of muscle through monocarboxylate transporter 4 (MCT4) into the bloodstream to be carried to the liver for gluconeogenesis (Dimmer et al., 2000). Glucose is then stored in the liver as glycogen or circulated as glucose, which can be consumed by skeletal muscle in a process termed the Cori Cycle. Most reports agree gene expression and protein abundance of LDHα decreases in WB muscle (Kuttappan et al., 2017a; Malila et al., 2019; Thanatsang et al., 2020; Zhao et al., 2020; Wang et al., 2023), which corresponds to the decrease in lactate. Contradicting reports indicate no difference (Zhao et al., 2020) or an increase in protein abundance in WB muscles (Soglia et al., 2016); however, the latter report did not indicate the LDH subunit evaluated. Lactate dehydrogenase beta (LDHβ) converts lactate to pyruvate (Cahn et al., 1962; Dawson et al., 1964; Pesce et al., 1964), and LDHβ gene expression as well as protein abundance increased 8.4- and 13.6- fold in WB muscles compared to unaffected PM muscles, respectively (Zhao et al., 2020; Wang et al., 2023). Interestingly, Zhao et al., 2020 reported no differences in protein abundance of monocarboxylate transporter 1 (MCT1; facilitates lactate uptake into cells), while protein abundance of MCT4 (facilitates lactate export) increased 3.4-fold in WB muscles compared to unaffected PM muscles. In osteosarcoma cells, MCT4 is upregulated during hypoxic conditions, which is partially attributed to a HIF-1α mediated mechanism and may be employed by cells to compensate for an increase in lactate production (Sheng et al., 2023). Although an increase in MCT4 would normally suggest an increase in lactate production and efflux from myofibers feeding into the Cori Cycle, this does not appear to be the case in WB muscle. Collectively, these reports suggest WB muscles still employ mechanisms to accommodate anaerobic conditions; however, glycolytic flux and lactate production are obstructed (Figure 2).

#### 2.1.2 Pentose phosphate pathway

Interestingly, there is little evidence that indicates impaired glucose uptake is responsible for the diminished glycolytic flux, which questions if all metabolic pathways associated with glycolysis are also impaired in WB muscle. For example, G-6-P may be diverted into the PPP to support NADP^+^ reduction to replenish NADPH pools, which is necessary for fatty acid synthesis and reduction of glutathione to scavenge ROS (Stincone et al., 2015). In addition to hypoxia, oxidative stress is also a hallmark of WB. The notion that WB muscles divert glucose into the PPP to replenish NADPH pools is supported by an increase in hexokinase-1 protein abundance in WB muscles (Kuttappan et al., 2017a), which indicates an increase in glucose phosphorylation to G-6-P. Although G-6-P levels are lower in WB muscles, it is possible G-6-P is being diverted into the PPP to restore a diminishing NADPH pool. Indeed, levels of the PPP intermediates 6-phosphogluconate and sedoheptulose 7-phosphate are 2.84 - and 3.73- fold higher in WB muscles compared to unaffected PM muscles, respectively (Abasht et al., 2016). Further, cytidine, thymidine, adenine, and guanosine levels increase in WB (Abasht et al., 2016), which are synthesized using ribose-5-phosphate from the PPP. There is also evidence of increased nucleotide catabolism in WB (Abasht et al., 2016), which is associated with a mitochondrial response to oxidative stress (Kristal et al., 1999). Therefore, it is possible the WB pathology forces muscle to shunt glucose into the PPP to combat oxidative stress at the expense of glycolytic capacity (Figure 2).

#### 2.1.3 Serine biosynthesis pathway

In addition to an increase in PPP diverting glucose from ATP production, the serine biosynthesis pathway (SBP) may also shunt glycolytic intermediates to support additional glutathione synthesis to combat oxidative stress. Glutamate, cysteine, and glycine are amino acid precursors for glutathione synthesis. Glycine as well as cysteine are synthesized from serine, which is produced from the glycolytic intermediate 3-phopshoglycerate. In fact, gene expression of the enzyme that catalyzes the first step of serine biosynthesis, phosphoglycerate dehydrogenase (PHGDH), is upregulated 11.6-fold in WB muscle compared to unaffected PM muscles (Mutryn et al., 2015). Further, gene expression of the enzyme that catalyzes the first step of glutathione synthesis is also upregulated in WB muscle (Zhang et al., 2023). This notion is supported by an increase in cysteine, glutathione, and oxidized glutathione levels in WB muscles (Abasht et al., 2016; Thanatsang et al., 2020) as well as an increase in gene expression and protein abundance of several enzymes involved in glutathione-mediated scavenging in WB muscles (Figure 2) (Carvalho et al., 2023; Zhang et al., 2023). These findings support the notion that glycolytic intermediates are being diverted into metabolic pathways to combat stress.

#### 2.1.4 Hexosamine biosynthetic pathway

While it is apparent WB muscle employs metabolic pathways to counteract oxidative stress, it appears this occurs at the expense of ATP production to meet cellular energy demands. Indeed, ATP levels are decreased in WB muscle compared to unaffected PM muscles (Baldi et al., 2020). Given PM muscles are inherently glycolytic and commercial broilers are fed high energy diets, a potent underlying mechanism must be obstructing glycolytic capacity in WB. Interestingly, feeding low energy or low protein diets decreases the prevalence of myopathies in growing broilers (Trocino et al., 2015; Meloche et al., 2018b; Meloche et al., 2018a; Simoes et al., 2020; Vieira et al., 2021). While this may be attributed to a diet-induced slower growth rate, high energy diets may force muscle into a state of metabolic disease. In fact, UPD-GlcNAc levels are higher in WB muscles compared to unaffected PM muscles (Abasht et al., 2016), which is the precursor for the nutrient sensitive post-translational modification O-GlcNAcylation, and confirms WB muscle still responds to the high energy diet. This is a possible mechanism employed by WB muscle to divert glycolytic metabolites into the PPP or other pathways branching from glycolysis (Figure 2). This notion is supported by reports of O-GlcNAcylation increasing hexokinase-1 activity as well as glucose-6-phosphate dehydrogenase (Rao et al., 2015; Baldini et al., 2016; Liu et al., 2022), which catalyzes the first step of the PPP. Therefore, it is possible the high nutritional plane combined with an expedited growth rate culminate in metabolic conditions that signal a state of excess nutrients to “override” stress responses during the onset and progression of disease. However, such a mechanism has yet to be identified but further investigation into the regulation of glycolysis and its ancillary pathways will shed light on the unique metabolic fingerprint of WB muscle.

### 2.2 Mitochondrial metabolism in WB muscle

Alternative to its use in the Cori Cycle, pyruvate can be shuttled into mitochondria to enter the TCA cycle through PDH or PC. In addition, the TCA cycle can metabolize amino acids as well as acetyl-CoA from fatty acid beta-oxidation as alternative metabolite sources to carbohydrates. In highly glycolytic type IIB fibers, ATP production largely occurs through glycolysis, and thus mitochondrial metabolism is a minor contributor to cellular ATP production (Kunz, 2001). Indeed, mitochondrial content is low in type IIB fibers of PM muscles compared to more oxidative fiber types of broiler gastrocnemius muscles (Hosotani et al., 2021). Mitochondria in type IIB fibers of PM muscles are small, dispersed, and ellipsoid-shaped compared to elongated and interconnected mitochondrial networks found in oxidative fibers of gastrocnemius muscles (Hosotani et al., 2021). Although mitochondrial content in healthy PM muscles is low compared to more oxidative fiber types, PM mitochondria undergo fusion and fission events to preserve mitochondrial function, albeit in a more tempered manner than oxidative fibers (Hosotani et al., 2021). Mitochondrial dynamics are perturbed in WB muscle contributing to swollen mitochondria morphology, an increase in ROS production, and overall metabolic dysregulation (Hosotani et al., 2020; Hasegawa et al., 2022; Carvalho et al., 2023; Wang et al., 2023).

#### 2.2.1 Metabolite utilization in the TCA cycle

Although the TCA cycle is often illustrated as a continuous cycle of enzymatic reactions, there are several entry and exit points within the cycle that enable mitochondria to accommodate cellular energy demands. The TCA cycle is not a carbon sink, and therefore an equilibrium exists between metabolites feeding into the TCA cycle and its intermediates exiting the cycle. These processes are termed anaplerosis and cataplerosis, where the former are a series of enzymatic reactions replenishing the pool of TCA intermediates and the latter remove TCA intermediates from the cycle. While anaplerosis (metabolites entering the TCA cycle) is often the center of discussion, cataplerosis (intermediates exiting the cycle) is often overlooked. Cataplerotic reactions are essential for regulating glucose, amino acid, and fatty acid metabolism. For example, in the glycolytic type IIB fibers of PM muscles, carbohydrate-derived metabolites are the predominate fuel source used in glycolysis, but pyruvate can also feed into the TCA cycle and then exit at several points to support amino acid synthesis. These cataplerotic reactions are necessary to support rapid protein accretion to enable the fast rate of muscle hypertrophy observed in commercial broilers. However, mitochondrial dysfunction in WB is presumably a rate limiting factor in successfully accommodating the high demands of amino acid metabolism in these fast-growing broilers.

**FIGURE 3:**
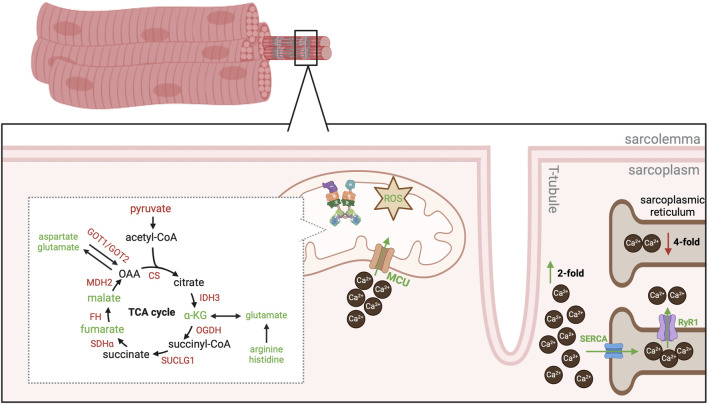
Overview of mitochondrial metabolism in WB muscle. Schematic of enzymatic and intermediate changes in the TCA cycle and associated cataplerotic / anaplerotic reactions. In addition, changes in calcium handling are also depicted. Red text or arrows indicates a decrease in either gene expression or protein abundance of the enzyme orintermediate. Green text or arrows indicates an increase in either gene expression or protein abundance of the enzyme or intermediate. Black text was either not evaluated or no changes were reported. Abbreviations: CS, citrate synthase; IDH3, isocitrate dehydrogenase 3; α-KG, alpha-ketoglutarate; OGDH, oxoglutarate dehydrogenase; SUCLG1, succinyl-CoA ligase; SDHα, succinate dehydrogenase alpha; FH, fumarate hydratase; MDH2, malate dehydrogenase 2; GOT1, glutamic-oxaloacetic transaminase 1; GOT2, glutamic-oxaloacetic transaminase 2; ROS, reactive oxygen species; MCU, mitochondrial calcium uniporter; SERCA, sarcoendoplasmic reticulum calcium ATPase; RyR1, ryanodine receptor 1.

A decrease in gene expression and protein abundance of most TCA cycle enzymes is observed in WB muscle (Kuttappan et al., 2017a; Papah et al., 2018; Carvalho et al., 2023; Wang et al., 2023). Interestingly, levels of α-ketoglutarate, fumarate, and malate increase in WB muscle but there is little evidence of changes in other TCA intermediates (Figure 3) (Abasht et al., 2016; Greene et al., 2020). These three intermediates are all involved in amino acid metabolism and may be an attempt for WB muscle to accommodate protein synthesis rate. For example, glutamate dehydrogenase catalyzes the reversible conversion of α-ketoglutarate to glutamate, which facilitates use of glutamate as a fuel source during times of energy scarcity or synthesis of glutamate during times of energy surplus (Plaitakis et al., 2017). As levels of glutamate are elevated in WB muscle (Greene et al., 2020; Wang et al., 2023) and it is a precursor for glutathione synthesis, this may be another point of diversion for metabolites to combat oxidative stress in WB muscle. However, a decrease in gene expression and protein abundance of isocitrate dehydrogenase 3 (Kuttappan et al., 2017a; Wang et al., 2023), the enzyme that catalyzes the oxidative decarboxylation of isocitrate to α-ketoglutarate, as well as a decrease in citrate synthase activity (Figure 3) (Li et al., 2022; Li et al., 2024) suggests the increase in glutamate is not attributed to carbohydrate metabolism but rather another metabolite source. An increase in histamine and arginine levels increase in WB muscle may be supporting glutamate synthesis but it remains ambiguous (Figure 3) (Abasht et al., 2016). These reports indicate mitochondria also divert metabolites to combat oxidative stress, although the intricacies of skeletal muscle metabolism make it difficult to elucidate the mechanisms of nutrient utilization in WB.

In addition, fumarate and malate levels are elevated in WB muscle (Abasht et al., 2016; Greene et al., 2020), although the biological implications of an increase in these intermediates is also unclear. For example, a decrease in abundance of fumarate hydratase (catalyzes the reversible conversion of malate to oxaloacetate) and malate dehydrogenase 2 (catalyzes the reversible conversion of malate to oxaloacetate) (Kuttappan et al., 2017a; Carvalho et al., 2023; Wang et al., 2023) but increase in fumarate and malate is counterintuitive (Figure 3). Further, cataplerotic enzymes that divert fumarate and malate out of the TCA cycle are also decreased in WB muscle. In healthy muscle, malate can be converted to oxaloacetate and used by glutamic-oxaloacetic transaminase 1 or 2 (GOT1 or GOT2) to generate aspartate or glutamate. However, a decrease protein abundance of GOT1 and GOT2 but an increase in aspartate levels in WB muscle is perplexing (Figure 3) (Abasht et al., 2016; Kuttappan et al., 2017a; Greene et al., 2020; Wang et al., 2023). Therefore, it is unclear if these intermediates are playing an important role in an unidentified metabolic process or if TCA intermediates “bottleneck” as fumarate and malate. Regardless, it is clear mitochondrial metabolic pathways are dysregulated in WB muscle.

#### 2.2.2 Mitochondrial calcium handling

The obscurity of metabolite utilization makes it difficult to define the underlying cause of metabolic dysregulation in WB muscle. The metabolic fingerprint of WB muscle appears to be a unique combination of combating oxidative stress and responding to a state of excess nutrients as well as hypertrophic stimuli. Although the exact mechanism is undefined, an increase in cytoplasmic calcium (Ca^2+^) and mitochondrial Ca^2+^ uptake provokes muscle hypertrophy (Klont et al., 1994; Scheffler et al., 2014; Mammucari et al., 2015; Gherardi et al., 2019); however, Ca^2+^ overload induces apoptosis and disease (Gommans et al., 2002). Cytoplasmic Ca^2+^ levels are 2- fold higher and sarcoplasmic reticulum (SR) Ca^2+^ levels are 4- fold lower in WB muscle compared to unaffected PM muscles suggesting either a leaky SR or impaired SR Ca^2+^ uptake (Zhang et al., 2023). Abundance of the sarcoendoplasmic reticulum calcium ATPase (SERCA) pump is markedly higher in WB muscle compared to unaffected PM muscles (Soglia et al., 2016; Zhang et al., 2023), which points to a defect in Ca^2+^ release. Indeed, gene expression of the Ca^2+^ release channel ryanodine receptor 1 (RyR1) is higher in WB muscle (Zhang et al., 2023) as well as protein abundance of the chloride intracellular channel protein (Carvalho et al., 2023), which is a negative regulator of RyR1-mediated Ca^2+^ release. These reports suggest leaky Ca^2+^ release from the SR and an attempt to curb RyR1-mediated Ca^2+^ release (Figure 3). In aging human models, excessive and prolonged exposure to ROS results in irreversible damage of RyR1 (Fulle et al., 2004). As mitochondrial ROS generation is higher in WB muscle compared to unaffected PM muscles (Zhang et al., 2023), these reports question if impaired Ca^2+^ handling observed in WB muscle is attributed to selection for exacerbated muscle hypertrophy or ROS inducing SR damage. Regardless, it is clear the increase in cytoplasmic Ca^2+^ results in greater mitochondrial Ca^2+^ uptake (Zhang et al., 2023).

An increase in mitochondrial calcium uniporter gene expression and protein abundance confirms WB mitochondria attempt to buffer high cytoplasmic Ca^2+^ levels (Zhang et al., 2023). Mitochondrial metabolism is stimulated by Ca^2+^ uptake, which is thought to be a mechanism to match energy supply and energy demand in a process termed parallel activation (McCormack and Denton, 1981). Of note, Ca^2+^ stimulates ATP synthase to increase oxidative phosphorylation and ATP production (Jouaville et al., 1999). An increase in myoglobin gene expression and protein abundance supports the notion of WB muscle attempting to increase oxidative capacity (Mutryn et al., 2015; Carvalho et al., 2023). However, recent evidence also suggests Ca^2+^ binds to F-ATP synthase β subunit (ATP5β), which provokes conformational changes to induce opening of the mitochondrial permeability transition pore (mPTP) and eventually apoptosis (Giorgio et al., 2017). An increase in ATP5β abundance (Carvalho et al., 2023; Zhang et al., 2023) but decrease in mitochondrial respiratory capacity in WB muscle (Zhang et al., 2023) suggests a Ca^2+^ mediated mechanism is partially responsible for mitochondrial dysfunction in WB.

## 3 Postmortem metabolism and fresh meat quality attributes in WB meat

The poor quality of WB meat continues to cost the U.S. poultry industry millions of dollars annually (Kuttappan et al., 2016; Huang and Ahn, 2018). For example, WB meat has a poor visual appearance, high drip loss, low marinade uptake, high cook loss, and inferior tenderness when compared to non-affected filets (Mudalal et al., 2015; Soglia et al., 2016; Soglia et al., 2017). In fact, over 50% of consumers indicated they would not buy moderately or severely affected WB filets (Kuttappan et al., 2012), which forces processors to downgrade WB products (Kuttappan et al., 2012; Petracci et al., 2013). While the development of fresh meat quality is a multifactorial process, several defects incurred from the myopathy contribute to the poor eating experience associated with WB meat.

Severe myodegeneration with partial myofibrillar regeneration in WB muscle culminates in abnormal tissue structure and composition (Sihvo et al., 2014). In fact, an increase in protein degradation as well as diminished sarcomere organization and longer sarcomeres are routinely observed in WB muscle (Soglia et al., 2016; Kuttappan et al., 2017b; Papah et al., 2017; Baldi et al., 2020; Greene et al., 2020; Puolanne et al., 2021; Carvalho et al., 2023; Wang et al., 2023), which are features associated with diminished tenderness and water-holding capacity. Further, regions of damaged muscle that are not repaired fully exhibit an increase in immune cells, fibroblasts, and adipocytes indicating WB muscle coordinate an attempt to repair the tissue, but non-muscle cells infiltrate the damaged tissue (Figure 1) (Sihvo et al., 2014; Sihvo et al., 2017; Ferreira et al., 2020; Andretta et al., 2021; Ziemkiewicz et al., 2021). Indeed, thickening of the endo- and perimysium connective tissue layers as well as an increase in total collagen content, heat-insoluble collagen, and adiposity occurs in WB muscle (Soglia et al., 2016; Geronimo et al., 2022; Li et al., 2023). These structural and compositional changes contribute to an increase in tensile strength and the hard palpable areas found in WB filets (Sihvo et al., 2014; Sihvo et al., 2017; Ferreira et al., 2020; Andretta et al., 2021; Ziemkiewicz et al., 2021). While these physiochemical characteristics contribute to an inferior meat product, the underlying biochemical changes also play a role in altered water holding capacity, textural characteristics, and processing abilities (Petracci et al., 2013; Kuttappan et al., 2017b; Sihvo et al., 2017; Bowker et al., 2018; Dalgaard et al., 2018).

### 3.1 Postmortem metabolism

The metabolic dysregulation observed in living WB muscle translates to altered postmortem metabolism during the conversion of muscle to meat. For example, high ultimate pH is one of the hallmark characteristics of WB meat (Sihvo et al., 2014; Mutryn et al., 2015; Soglia et al., 2016; Kuttappan et al., 2017b; Sihvo et al., 2017; Soglia et al., 2017; Baldi et al., 2019; Tasoniero et al., 2019; Baldi et al., 2020; Li et al., 2023; Li et al., 2024). Under normal conditions, high ultimate pH is typically associated with an increase in water-holding capacity; however, water-holding capacity is diminished in WB meat. This may be attributed to the structural changes observed in WB muscle, such as an increase in sarcomere length (Baldi et al., 2020; Puolanne et al., 2021), disorganized sarcomere structure (Papah et al., 2017), and an increase in protein degradation. Further, greater adiposity and collagen content in WB also contributes to poor water-holding capacity because muscle plays a major role in binding water during storage and processing. Therefore, defects in postmortem metabolism are not the sole contributor to poor meat quality in WB; however, biochemical changes in muscle play a role in fresh meat quality and understanding these changes may contribute to the development of intervention strategies to salvage WB meat.

In WB muscle, glycogen stores and enzymes involved in glycogen metabolism are reduced compared to unaffected PM muscles (Abasht et al., 2016; Kuttappan et al., 2017a; Baldi et al., 2020; Carvalho et al., 2023). As glycogen availability is a major factor dictating pH decline (Henckel et al., 2000; Immonen et al., 2000a; Immonen and Puolanne, 2000; Immonen et al., 2000b; Chauhan and England, 2018; Chauhan et al., 2019; Spires et al., 2023), this seemed to be the culprit for an abbreviated pH decline. However, Baldi et al., 2020 reported residual glycogen was present at 24 h, which suggests depleted glycogen stores are not responsible for arresting glycolysis prematurely in WB meat. In the presence of residual glycogen, activity of phosphofructokinase (PFK) is a key player in determining ultimate pH (Matarneh et al., 2018b), but PFK activity early postmortem is not different in WB suggesting PFK is not responsible for the high ultimate pH (Baldi et al., 2020). However, glycolytic capacity of WB meat is diminished (Baldi et al., 2020; Li et al., 2024), which questions if the biochemical changes observed in WB muscle impact postmortem metabolism. For example, WB muscle appears to divert glycolytic intermediates through ancillary pathways to combat oxidative stress through downregulation of many glycolytic enzymes, which may culminate in reduced glycolytic capacity and high ultimate pH in WB meat. This notion is supported by reduced levels of glucose and nearly undetectable levels of G-6-P at 24 h postmortem in WB meat (Baldi et al., 2020). Further, WB muscle downregulates LDHα and WB meat exhibits a decrease in lactate accumulation (Abasht et al., 2016; Kuttappan et al., 2017a; Malila et al., 2019; Baldi et al., 2020; Thanatsang et al., 2020; Zhao et al., 2020; Wang et al., 2023; Li et al., 2024). Although these metabolic changes have not been entirely defined, it is clear that changes in WB muscle impact postmortem metabolism in WB meat.

After exsanguination, and thus removal of oxygen supply, mitochondria retain functionality using oxygen stores provided by myoglobin postmortem (England et al., 2018). Although glycolysis unarguably drives postmortem metabolism, mitochondria are not obsolete during this process and contribute to pH decline (Scheffler et al., 2015; England et al., 2018; Matarneh et al., 2018a; Matarneh et al., 2018b). In fact, addition of mitochondria to an *in vitro* system that mimics postmortem metabolism alters the utilization of the glycolytic end product pyruvate (Matarneh et al., 2021). Further, addition of mitochondria increases ATP hydrolysis and thus glycolytic flux in this *in vitro* system; however, inhibition of ATP5β negates the mitochondrial enhancement of glycolytic flux (Matarneh et al., 2018a). In WB muscle, protein abundance of ATP5β is greater than unaffected PM muscle (Carvalho et al., 2023; Zhang et al., 2023), which would suggest an increase in ATP hydrolysis and glycolytic flux. However, mitochondrial metabolic dysregulation and decreased respiratory capacity (Kuttappan et al., 2017a; Papah et al., 2018; Carvalho et al., 2023; Wang et al., 2023; Zhang et al., 2023) may negate the contribution of WB mitochondria to postmortem metabolism.

### 3.2 Postmortem proteolysis

Tenderness is determined by many factors including connective tissue characteristics, myofibrillar structure, sarcomere length, protein degradation, and intramuscular fat. An increase in shear force and tensile strength is often observed in WB meat (Chatterjee et al., 2016; Soglia et al., 2017; Hasegawa et al., 2020; Jarvis et al., 2020), which supports the notion that tenderness deteriorates in WB meat. In normal tissue, high free Ca^2+^ is associated with an increase in postmortem proteolysis and tenderness through calpain activation (Wheeler et al., 1992; Boleman et al., 1995; Dang et al., 2020). Although free Ca^2+^ is elevated in WB (Soglia et al., 2016; Tasoniero et al., 2019; Welter et al., 2022; Wang et al., 2023), calpain activation as well as proteolytic degradation of desmin and troponin T has been reported as either not different or increased in WB meat compared to unaffected filets (Soglia et al., 2017; Soglia et al., 2018; Hasegawa et al., 2020; Welter et al., 2022). Although there is not a consensus on whether calpain activation is unchanged or increased in WB meat, these reports agree that defects in postmortem proteolysis are not at fault in WB meat. However, changes in sarcomere length and structure (Papah et al., 2017; Baldi et al., 2020; Puolanne et al., 2021), altered protein composition (Baker et al., 1999; Soglia et al., 2016; Kuttappan et al., 2017a; Greene et al., 2020; Carvalho et al., 2023; Wang et al., 2023), and higher total collagen content as well as increased heat-insoluble collagen (Soglia et al., 2016; Geronimo et al., 2022; Li et al., 2023) in WB myofibers are presumably large drivers of poor tenderness in WB meat. In fact, Hasegawa et al., 2020 reported tensile strength remained high with minimal changes to connective tissue in WB meat between harvest and 5 days postmortem. While the exact mechanism responsible for diminishing tenderness of WB meat remains vague, these reports suggest defects in postmortem proteolytic process are not driving tenderness deterioration in WB meat.

## 4 Conclusion

Wooden breast is a complex myopathy that not only affects the structural and metabolic characteristics of muscle, but also culminates in poor fresh meat quality attributes. For example, WB muscle appears to shunt glycolytic intermediates into ancillary pathways of glycolysis to combat oxidative stress through downregulation of many glycolytic enzymes, which presumably contributes to diminished glycolytic capacity and high ultimate pH of WB meat. Further, mitochondrial dysfunction may contribute to altered postmortem metabolism, but its exact impact on metabolism remains vague. Interestingly, postmortem proteolytic processes do not appear to be obstructed in WB meat, but high amounts of connective tissue, altered protein composition, and changes in sarcomere structure presumably drive tenderness deterioration in WB meat. In summary, metabolic dysregulation plays a role in the WB myopathy, but it remains unclear if changes in metabolism contribute to the onset of the disease or are simply a secondary symptom.
